# Supplementary material for: Heat Warnings in Switzerland: Reassessing the Choice of the Current Heat Stress Index
Source: Int J Environ Res Public Health. 2019 Jul 27;16(15):2684. doi: 10.3390/ijerph16152684 (PMC6696474; doi:10.3390/ijerph16152684)
Supplement: Supplementary file 1 [file ijerph-16-02684-s001.pdf]

International Journal of
Environmental Research and Public Health

MDPI

Supplementary

Heat warnings in Switzerland: Reassessing the choice of the current heat stress index

Annkatrin Burgstall, Ana Casanueva, Sven Kotlarski and Cornelia Schwierz

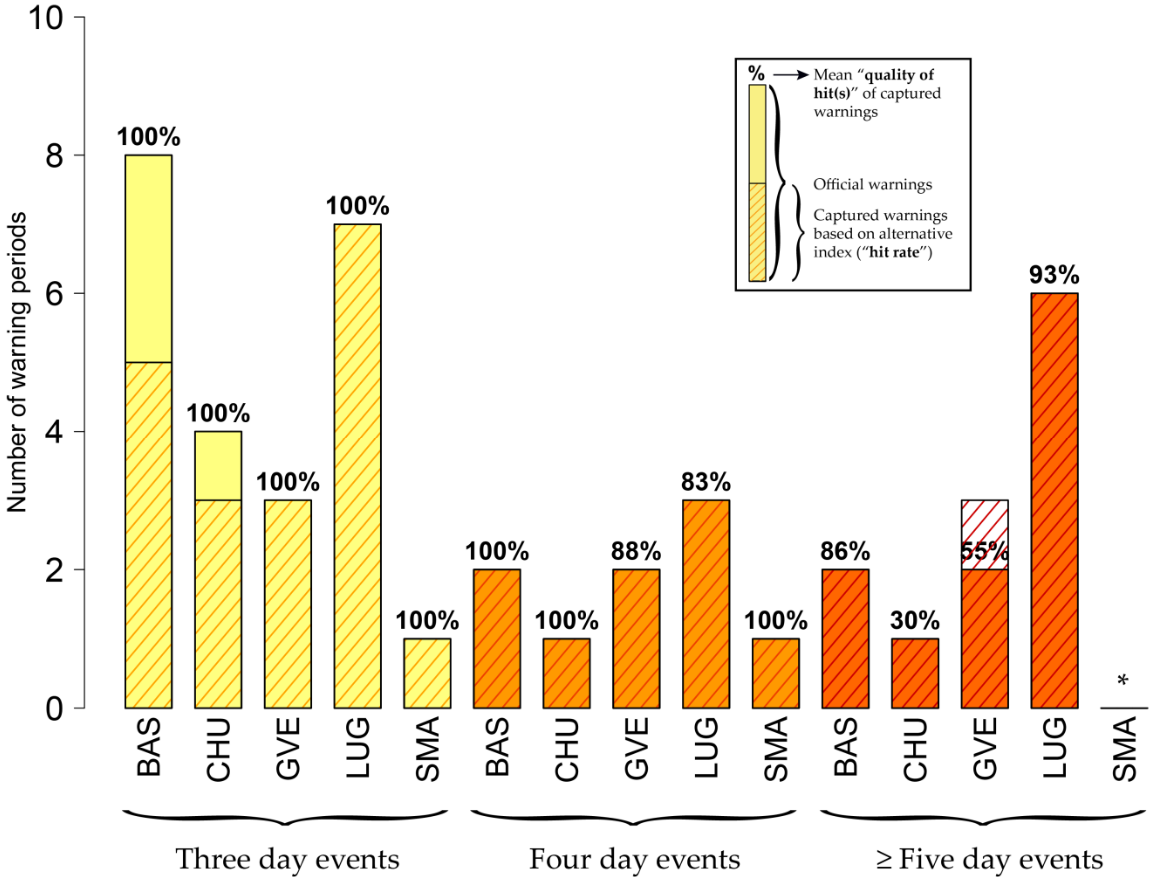

Figure S1. As Figure 10 but for *wbgt.shade*.

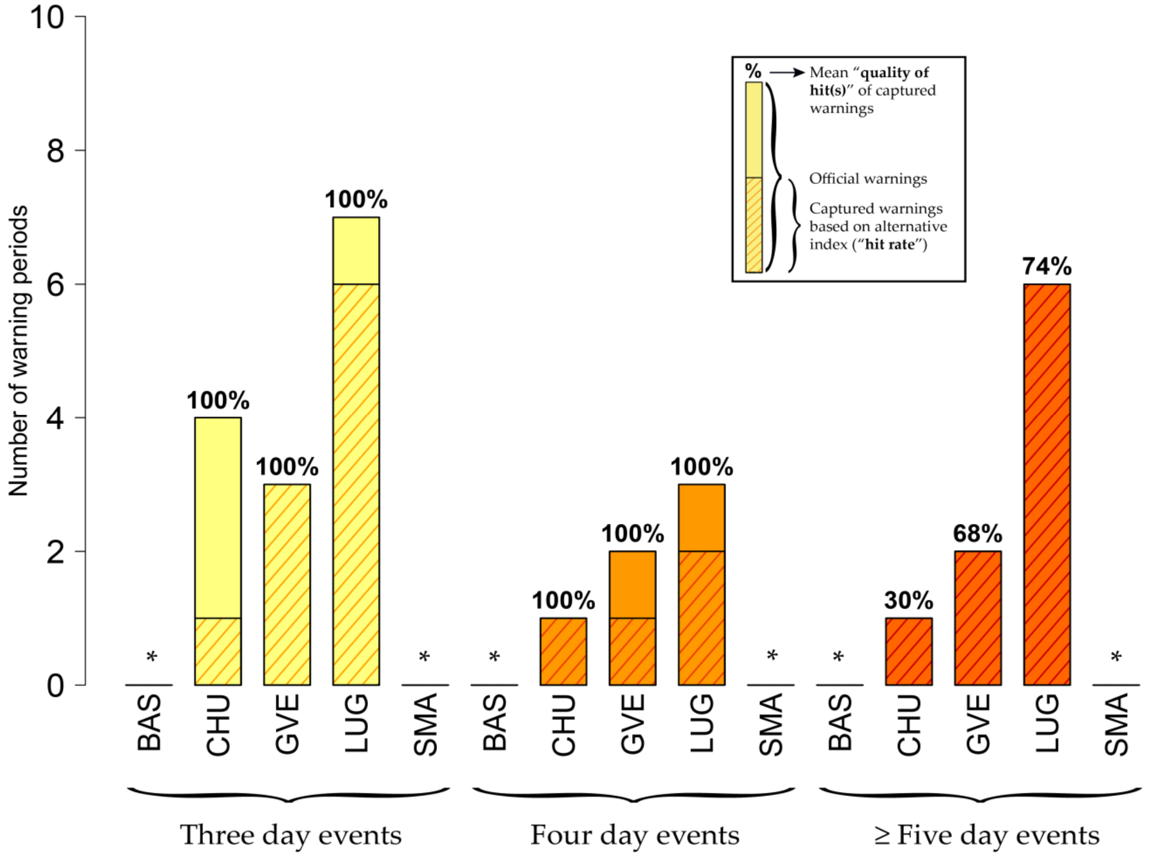

Figure S2. As Figure 10 but for *wbgt.sun*.

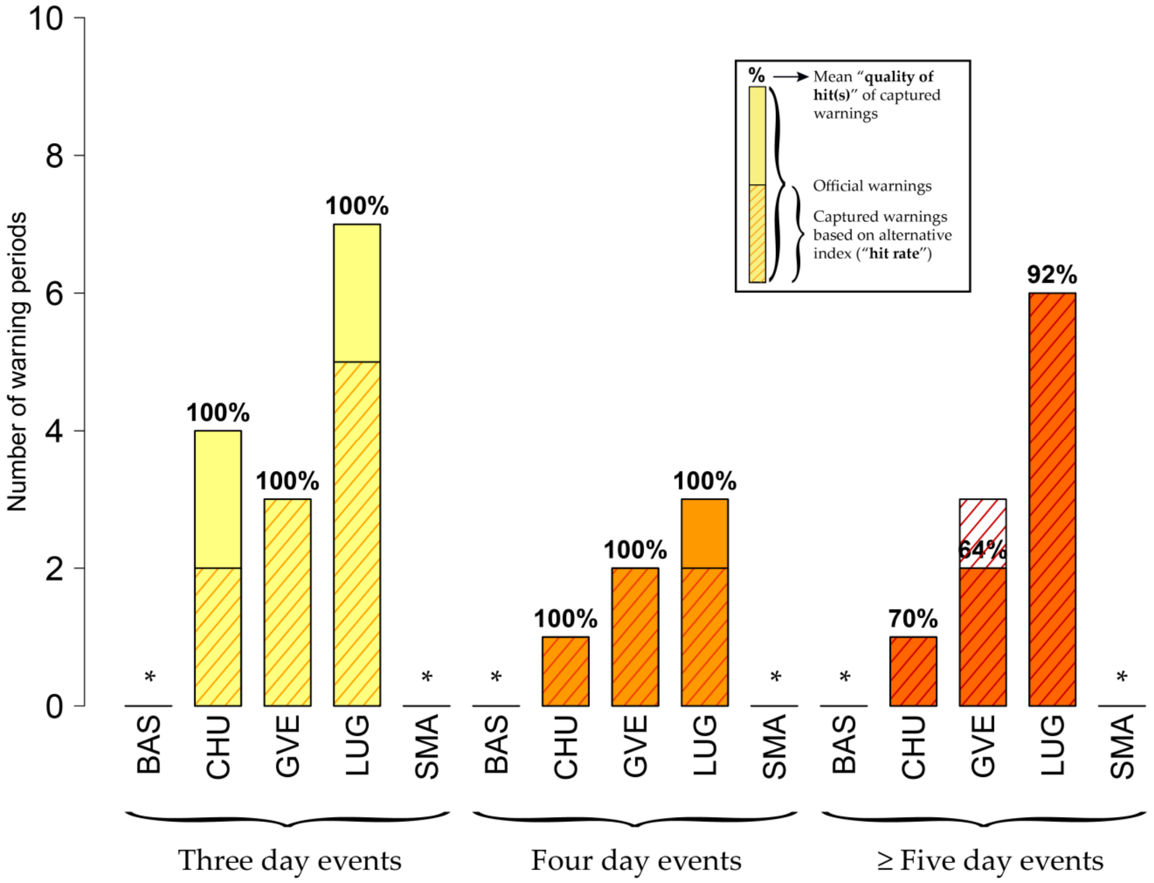

Figure S3. As Figure 10 but for *apparentTemp*.

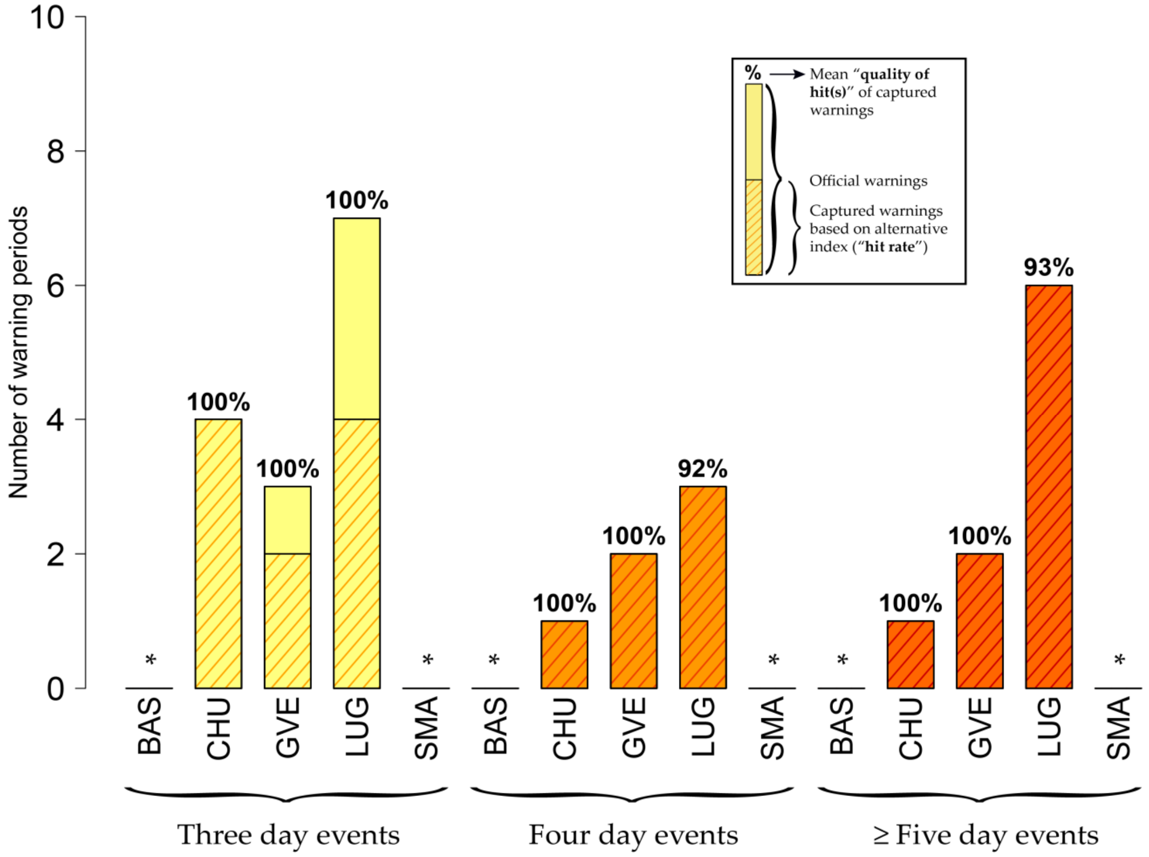

Figure S4. As Figure 10 but for *effectiveTemp*.

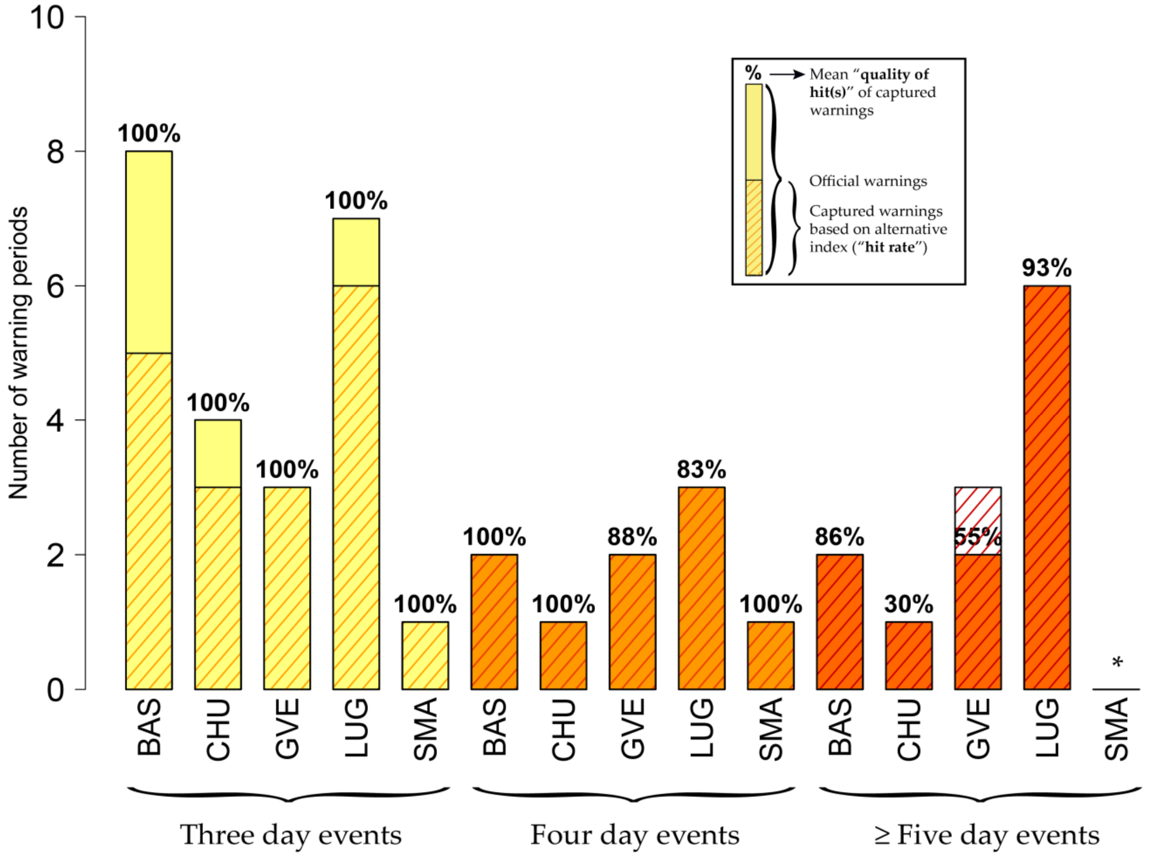

Figure S5. As Figure 10 but for *humidex*.

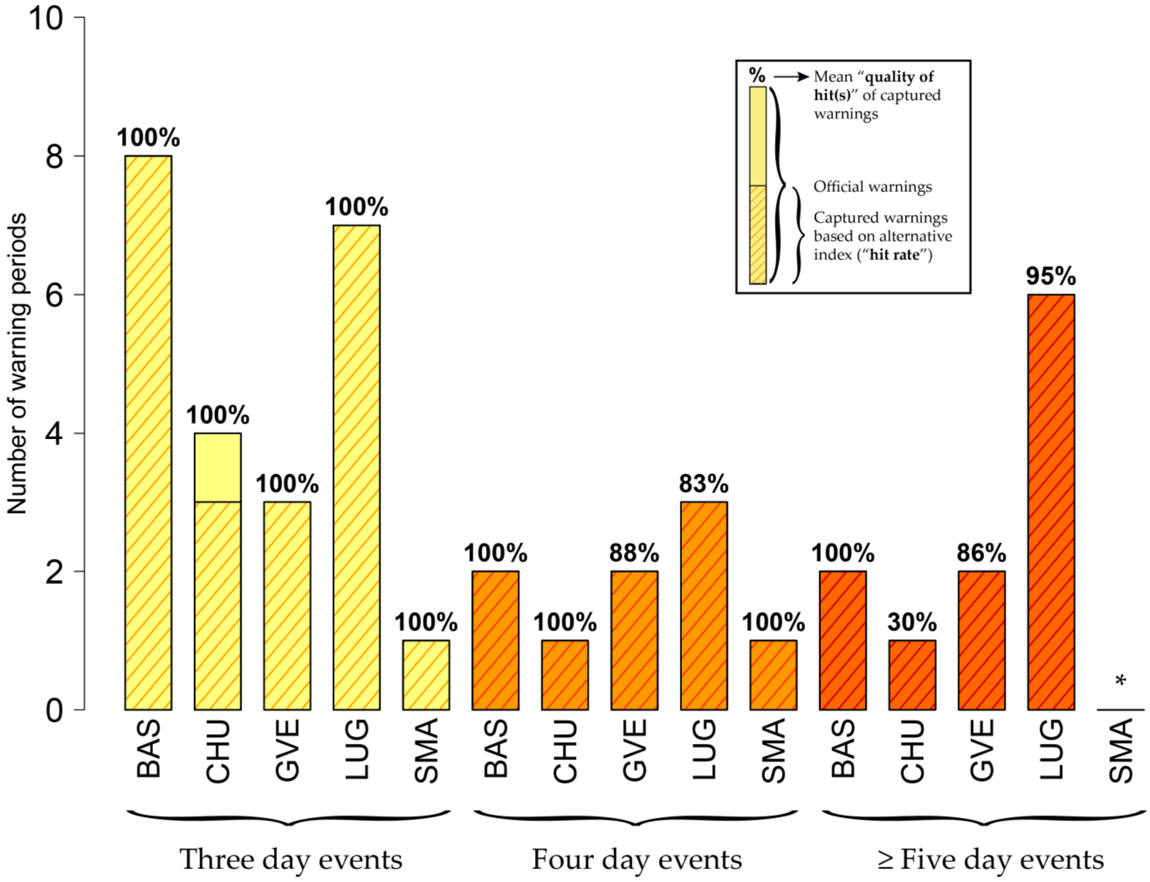

Figure S6. As Figure 10 but for *discomInd*.
